# Distinct Phenotype and Secondary Metabolite Profile Mark a Dominant *Aspergillus flavus* Outbreak Strain

**DOI:** 10.3390/jof12060454

**Published:** 2026-06-22

**Authors:** Alexander Gewecke, Noam Aviman, Jens C. Frisvad, Maiken Cavling Arendrup, Jakob Blæsbjerg Hoof

**Affiliations:** 1 Mycology Unit, Department for Bacteria, Parasites and Fungi, Statens Serum Institut, 2300 Copenhagen S, Denmark; ajkg@ssi.dk; 2 DTU Bioengineering, Technical University of Denmark, 2800 Kongens Lyngby, Denmark; noam.avim@gmail.com (N.A.); jcf@bio.dtu.dk (J.C.F.); 3 Department for Clinical Microbiology, Copenhagen University Hospital, Rigshospitalet, 2100 Copenhagen Ø, Denmark; 4 Department for Clinical Medicine, University of Copenhagen, 2100 Copenhagen Ø, Denmark

**Keywords:** *Aspergillus flavus*, aspergillosis, environmental sampling/environmental monitoring, surface microbiology, metabolomics, HPLC-DAD, mycotoxins, hospital outbreak/nosocomial outbreak, environmental contamination, outbreak

## Abstract

An *Aspergillus flavus* outbreak strain dominated the indoor environment in a Danish hospital ward for eight years and subsequently multiple isogenic infections occurred. We investigated whether strain-specific traits were present to understand its prevalence and persistence. The outbreak strain was studied alongside comparator *A. flavus* isolates with respect to altered virulence that could enhance its pathogenic potential and secondary metabolism that could influence environmental persistence. Twenty-four isolates were examined for growth patterns on ten media and by secondary metabolite profiling using high-performance liquid chromatography with diode-array detection. Strain-specific virulence and other phenotypic traits were studied in vivo using *Galleria mellonella* and in vitro by culturing on specialised media. No indication of virulence alterations was observed in larvae. However, the outbreak strain exhibited a reproducible fingerprint phenotype with distinct morphological features and secondary metabolites. These included mycotoxins known to be harmful to humans and animals. Although this study found no evidence of increased virulence, identification of a distinct phenotypic profile could indicate adaptation or an intrinsic ecological background of the outbreak strain and possibly competitive traits via potentially bioactive secondary metabolites. Moreover, the production of several mycotoxins by this *A. flavus* strain raises concerns for both patients and staff. Further analyses of the strain’s ecology, toxic potential, virulence, and phylogeny in a global context could be studied in future experiments.

## 1. Introduction

*Aspergillus flavus* is the second most common cause of invasive aspergillosis and of nosocomial *Aspergillus* outbreaks [1,2]. Beyond the clinical implications, identifying sources of contamination, whether from the external environment or within the hospital, and understanding how and why the fungus settles and persists in hospital environments remain challenging. These processes are difficult, expensive, and time-consuming to examine. From 2017 to 2025, a Danish tertiary hospital experienced an *A. flavus* outbreak mainly affecting children in a haematology ward, but also sporadic cases in non-haematologic patients and one case in the Department of Clinical Microbiology (DCM) following incubator contamination [3,4,5]. Retrospective analysis revealed a monophyletic cluster (Cluster-10) [5] with independent subbranches concurrently inhabiting and dominating each site. Genomics proposed the outbreak strain had been present in the hospital since 2008 [3,5], and that it was closely related to *A. flavus* isolates from commercially available building materials (wood-based) from an unrelated study [6]. That study had tested different materials for potential dormant mould spores and their germination after water damage [6]. Source investigation in the haematologic ward led to the discovery of extensive spore reservoirs on hard-to-reach surfaces, and indeed, direct growth of the outbreak strain on water-damaged plywood in a kitchen cabinet [5]. Clonal dissemination was ultimately linked to in-hospital construction work, a well-recognised route of dissemination for *Aspergillus* spores [2].

Following the outbreak, questions arose regarding virulence and why this particular type of strain was successful in persisting and outcompeting, e.g., other *A. flavus* strains in the outbreak ward. The answers may reflect its biological background. In a broader context, marked ecological versatility defines *A. flavus* as a saprotroph mould that thrives on decaying biological material, infects plants and animals, and contaminates crops such as maize and wheat, extending to annual crop losses worth billions of dollars [7]. This is largely due to the production of aflatoxins, insecticidal mycotoxins synthesised as secondary metabolites that enhance fungal survival in the field [8]. The toxin became notorious after the Turkey-X outbreak in the UK during the 1960s, in which approximately 100,000 turkeys died from severe hepatic damage following ingestion of the toxins [8]. Subsequent studies have shown both acute, chronic, and fatal cases of aflatoxicosis in humans following ingestion [7,9], with prolonged low-level exposure associated with immunosuppression and hepatocellular carcinoma [7]. Other common *A. flavus* secondary metabolites are potentially toxic to humans. For instance, cyclopiazonic acid inhibits the sarcoplasmic reticulum Ca^2+^ ATPase and shows adverse toxic effects in animals [10], and even small doses of aflatrem, found in sclerotia-producing strains, is neurotoxic in rats [11]. Furthermore, some strains of *A. flavus* can produce the highly neurotoxic 3-nitropropionic acid [12]. Little is known about the potential chronic mycotoxin exposure among hospitalised patients and healthcare staff when *Aspergillus* persists in hospital wards. However, a plausible hypothesis is that mycotoxins could worsen patient outcomes if produced during infection or tissue colonisation, or if present in the patient environment as a result of surface contamination or aerosolisation [13].

In addition to their toxic effects on mammals and insects, some secondary metabolites serve as chemical defences against other microbes. A famous example is penicillin production by the mould *Penicillium rubens*. Secondary metabolites also help *A. flavus* antagonise competing microorganisms via antimicrobials like aspergillic acid, aspirochlorin, and astellolides [14,15,16], and enable fungal persistence following oxidative stress via production of kojic acid, a potent antioxidant [17]. In addition, biological resilience and preservation mechanisms, through the formation of DNA and mycotoxin-packed sclerotia and conidia [18,19], enable *A. flavus* to survive elevated temperatures, low water activity, and nutrient deprivation [18]. It is not unreasonable to suggest that one or more of these listed factors enabled the outbreak strain to survive in the hospital’s interior.

Whether it has altered virulence in vivo, displays distinct morphological characters in vitro, or indeed produces harmful or specialised mycotoxins that may facilitate persistence and potentially endanger patients and staff is the focus of this study. The aims are to examine whether the outbreak strain possibly displays a specific phenotype profile and whether there is potential for ecological implications to any such features. As comparators, a panel of clinical and environmental *A. flavus* strains were selected based on phylogeny in our previous study [5]. We also added additional isolates from internal libraries to increase the volume of comparators for some of the analyses performed.

## 2. Materials and Methods

### 2.1. Strains and Isolates

A total of 24 isolates were included as summarised in Table 1.

Four main comparators were selected based on known genomic differences [5] to assess inter-strain virulence and phenotype differences to the outbreak strain. These differed from 16,825 to 50,462 SNPs from the outbreak strain “SS18” (Cluster-10). Isolate 8981 was previously identified tentatively as *A. flavus* by single medium morphology (yeast glucose chloramphenicol) [3], but could not be reliably placed in following phylogenetic analysis due to large divergence from other *A. flavus* genomes. It was nonetheless retained here as a known genetic outsider for phenotypic comparison. Strain NRRL 3357, previously used as genomic mapping reference [5], was included as a control (sourced from the Statens Serum Institut (SSI)). Aside from SS18, four additional Cluster-10 isolates from our previous study were selected to assess Cluster-10 phenotype variability. These differed by 82-86 SNPs (Wood1 to 3) and 76 SNPs (F16180, DCM patient’s isolate) compared to SS18. Additional outbreak ward surface isolates were included. Those were collected more recently from surfaces (N = 6) in the outbreak ward but had not been sequenced prior to this study. Lastly, external isolates with diverse origin (N = 9) were included from the Institut for Bioteknologi (IBT) Culture Collection at the Technical University of Denmark (DTU). These also included an IBT-version of the NRRL 3357 isolate (NRRL 3357 DTU). The two NRRL 3357 isolates (SSI/DTU) had been genotyped (see Table 1) prior to this study via short tandem repeats as described elsewhere [3]. All isolates were stored at −80 °C prior to use.

### 2.2. Galleria mellonella Virulence Assay

*G. mellonella* was used for virulence testing according to previous descriptions [20] with the following modifications. SS18 and the main comparators were incubated for three days at 37 °C prior to conidial harvest and suspension in PBS and concentration-adjustment to approximately 1 to 5 × 10^6^ using an optical density (OD) of 1 with verification of inoculum concentration by quantitative plating of serial dilutions. Three groups of 20 larvae for each strain were injected with 10 µL PBS solution of 1.5 × 10^6^ CFU/mL in a 5-fold dilution series hereof (5^−1^, 5^−2^, 5^−3^) using Hamilton syringes (Hamilton Company, Reno, NV, USA). PBS-injected and uninjected controls were included. Larvae were incubated at 37 °C for five days and monitored every 24 h for mortality. Dead larvae were removed upon recognition. Survival analysis was done in R.4.2.3 (package *survival*) and visualised in a Kaplan–Meier plot. Group survival was quantified via restricted mean survival time (RMST) [21]. A 2000 replicate bootstrap analysis of RMST estimates was employed to compare isolate-specific virulence. Benjamini–Hochberg (BH) adjustment was used to curb type I error.

Growth kinetic assays on solid agar at 37 °C had previously been performed for a Cluster-10 isolate and most of the main comparators (10822, F7734, 8981) with no difference observed.

### 2.3. Morphological and HPLC-DAD Profiling

Twenty-four isolates were cultured on different media made according to internal protocols (https://dtu.bio-aware.com/page/Cultivation%20media, URL (accessed on 14 June 2026) including *A. flavus* and *parasiticus* agar (AFPA), Creatine sucrose agar (CREA), Czapek Yeast Autolysate agar (CYA), Malt Extract agar (MEAox), Oatmeal agar (OAT), Sabouraud agar (SABO), Yeast extract sucrose agar (YES), Nakamura’s medium (MAN) [22], and Wickerham’s antibiotic test medium (WATM). Agar plates were three-point inoculated and grown in the dark for seven days at 25 °C (and at 37 °C for CYA specifically). For the morphological analysis, differences in colony diameter, degree of sporulation, pigment formation and acid production on CREA were assessed. Additionally, SS18 and the main comparators were inoculated on plywood-fibre agar (15 g fibre and 15 g agar per litre of water) at 37 °C for six days to test whether isolates could grow on that material or not. Growth on plywood was stained with Lactoethanol Cotton Blue (SSI Diagnostica, Hillerød, Denmark) prior to microscopy. Dichloran Glycerol agar (DG18) was also used to test xerotolerance among them.

For HPLC-DAD, plugs were extracted in pairs into a shared vial for CYA + YES, AFPA + MAN, MEAox + WATM, while single for SABO, from agar colonies following standardised procedures [23]. Secondary metabolites were separated via the Agilent 1100 system (Agilent Technologies, Waldbronn, Germany) with a Luna C18 column (100 × 2 mm, 3 µm) (Phenomenex, Torrance, California, USA). The mobile phase comprised water with 50 ppm trifluoroacetic acid and acetonitrile. Alkylphenone standards were included. Samples (3 µL) were analysed using the “Luna2ins.M” method on the Trubadurix platform. Detection by diode-array at 210 and 280 nm. Identification (IDs) were given to secondary metabolites via an internal DTU library based on retention indices (RI) of authentic standards by UV-visible (UV-VIS) spectra [23,24] and unique UV-VIS appearances. Additionally, a previous comparative study by Rank et al. 2012 [25] was used as reference for well characterised *A. flavus* and *A. oryzae* secondary metabolites. That study included metabolite identification by liquid chromatography coupled with tandem mass spectrometry (LC-MS/MS) of NRRL 3357 and RIB40 and was used to compare HPLC-DAD identified secondary metabolites found here. Results were reported as presence or absence of identified metabolites across media, and signal intensities of specific metabolites.

### 2.4. Genomic Basis of Secondary Metabolites

Illumina short read sequences of SS18 and the main comparators generated in our previous study [5] were compared using BLAST (National Library of Medicine) against query sequences of some commonly identified secondary metabolites from the NRRL 3357 reference (GenBank ac no.: GCA_009017415.1), RIB40 (GCF_000184455.2), and E1404 (GCA_013146025.1) [26,27] to further identify length and percentage similarity of gene clusters in the isolate-group. That was done via BLAST-assembly in CLC Main Workbench 25.0.3, sequence alignments, and pairwise gene cluster comparisons. The aim was to verify presence of the biosynthetic gene clusters (BGCs) for HPLC-DAD identified metabolites and compare the isolates in a broader context of *A. flavus* strains [26].

The internal transcribed spacer (ITS) and calmodulin (*CaM*) were compared to sequences of type cultures of the other members of the section *Flavi* by the multiple sequence alignment tool in CLC Main Workbench 25.0.3 to assign species identification for 8981 [28].

## 3. Results

### 3.1. Virulence Studies in the Galleria mellonella Virulence Assay

**Figure 1:**
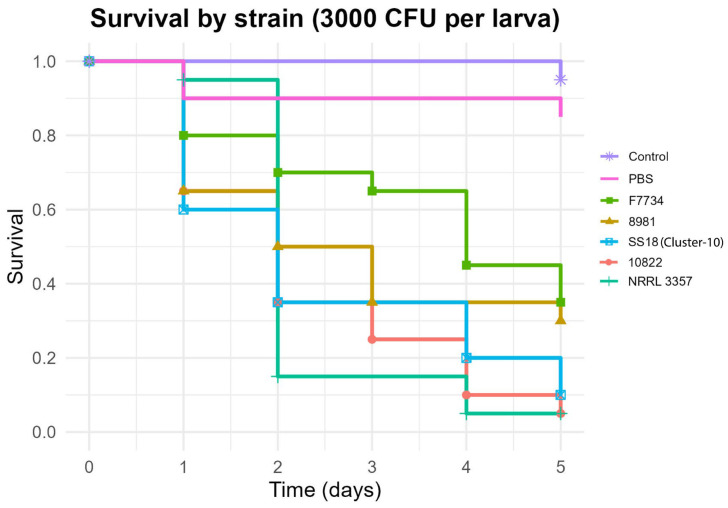
Kaplan–Meier survival. The plot displays comparable *G. mellonella* survival probability in percent over time (five days) after injection with SS18 or the main comparators. Most strains had killed 50% of the larvae after two days, except F7734, whereas 50% were dead after four days.

We used a larvae-based virulence assay to assess whether SS18 showed higher virulence than the main comparators Figure 1.

Concentration dependent (5^−1^, 5^−2^, 5^−3^) decrease in mortality of NRRL 3357-infected larvae was observed in a previous pilot study. The 5^−1^ injections remained potent for all strains, while mortality differences observed from 5^−2^ to 5^−3^ were minor. Therefore, only the 5^−1^ assay is presented here (Figure 1).

RMST survival comparison showed mean survival times of 2.30–3.60 days but the RMST difference in the bootstrap analysis among SS18 (Cluster-10) infected larvae was only significantly different (worse) when compared to the F7734 non-outbreak clinical isolate, but that difference was no longer significant after BH-adjustment. The differences in survival are presented as (days) deviation from the SS18 in Table 2.

### 3.2. Morphological Profiling

All isolates presented in this study were subjected to comprehensive One Strain Many Compounds analysis (OSMAC). This assay would reveal whether Cluster-10 isolates had an alternative morphotype and metabolic repertoire compared to an array of clinical and environmental isolates when exposed to a wide selection of conditions.

**Figure 2:**
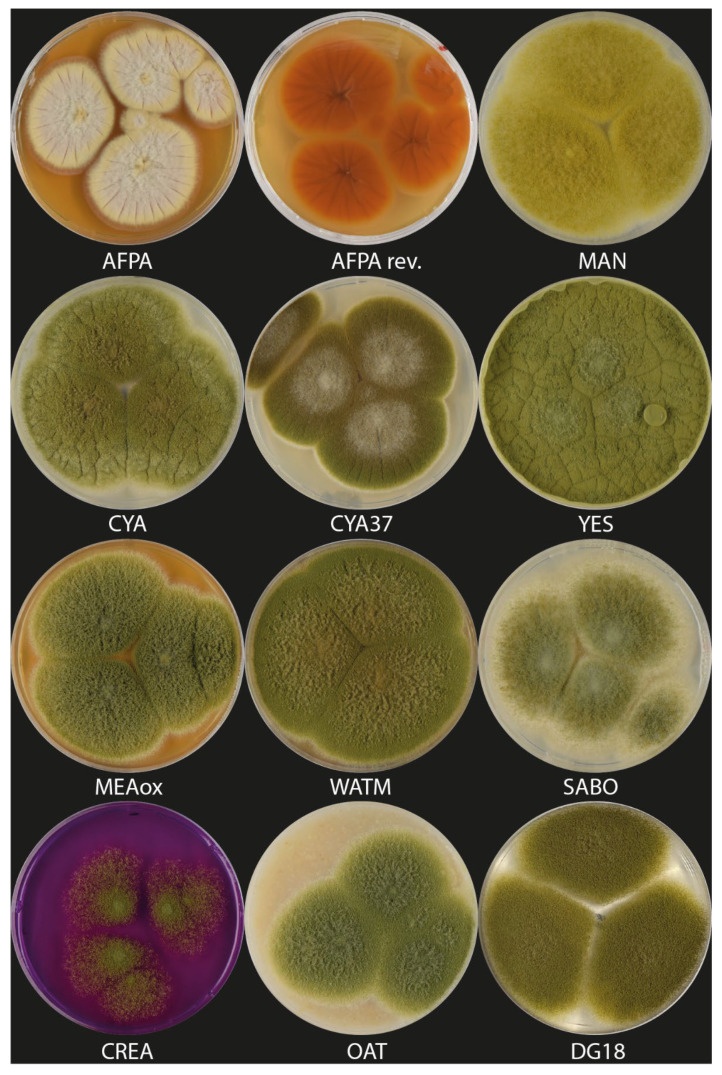
Common morphotype of Cluster-10 isolates. Growth on eleven media at 25 °C, 7 d (CYA37 at 37 °C, 7 d): AFPA: Colonies moderately deep, sulcate; mycelium white/cream; texture floccose; conidia absent; reverse bright orange. MAN: Colonies plane; mycelium white; texture floccose/powdery; conidia yellow abundant. CYA: Colonies moderately deep, sulcate; mycelium white; texture floccose centre, granular margin; conidia zonation from centre, brownish, olive-green, greyish-green, abundant; exudate droplets. CYA37 (37 °C, 7 d): Colonies plane, shallow sulci, good growth; mycelium white; texture floccose centre, powdery margin; conidia zonation from centre, brown, green, yellowish, abundant. YES: Colonies shallow/moderately deep, sulcate, lawn growth; texture granular/powdery; Heavy conidiation olive-green, darker-green; exudate large droplets. MEAox: Colonies thick; mycelium white, aerial hyphae; texture fluffy, moderate density; conidia olive-green, light-green, abundant. WATM: Colonies plane; mycelium white; texture floccose centre, powdery margin; conidia light-green, abundant towards margin. SABO: Colonies thick; mycelium white; texture fluffy, moderate/low density; conidia zonation from centre grey/greyish-green, moderate abundance. CREA: Poor growth; acid production absent. OAT: Colonies plane; mycelium white; texture floccose centre, powdery margin; conidia pale-green, abundant towards margin. DG18: Colonies plane, good growth; mycelium white; texture granular; conidia olive-green, abundant. Sclerotia were absent on all media.

Firstly, we established a common morphotype for Cluster-10, presented in a merged collection of the best images of Cluster-10 isolates that will serve as an overall ID of the lineage Figure 2.

**Figure 3:**
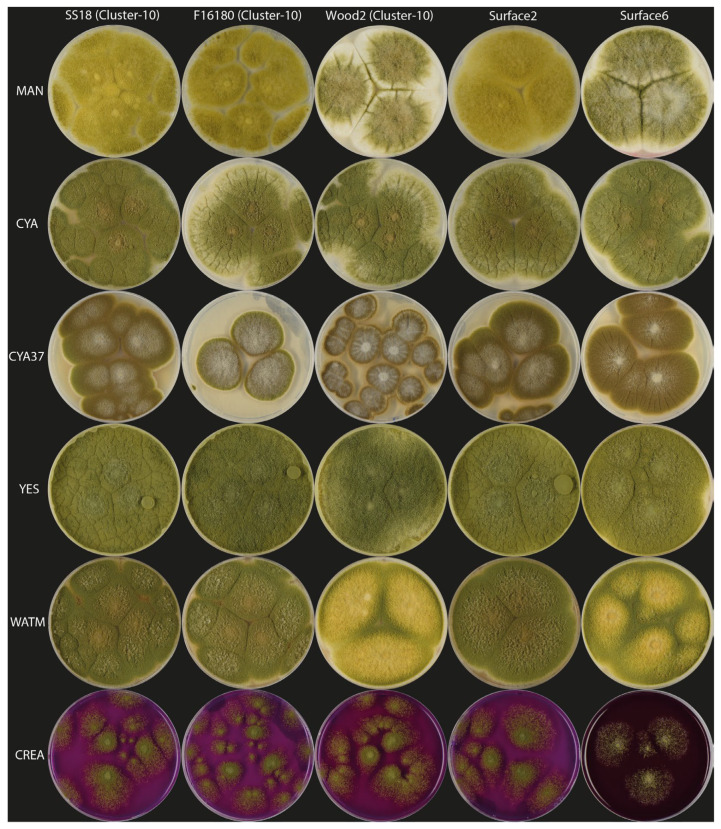
Morphological reproducibility among Cluster-10 and outbreak ward surface isolates. Cluster-10 showed a uniform morphology on most media including the clinical isolate F16180 from an infected DCM patient. A unique variation was shared among building material isolates (Wood1 to 3), and one isolate from an outbreak ward surface (Surface6) on the MAN-medium (sulcate, fluffy, greyish-green) and WATM (yellow with green margin). But the Surface6 isolate lacked exudate droplets on YES, although exudate was abundant for the building material isolates. That implies that isolates with both morphotype variations can be found inside the outbreak ward. Isolates not shown here can be found in Figure S1 in the Supplementary Files.

Among Cluster-10 isolates, the uniform morphotype was shared by all subbranches, although some variation was observed on MAN and WATM media; see Figure 3.

For SS18 and the main comparators, the most distinctive media were reported for comparison, while an overview of all media is provided in the Supplementary Files for all isolates in Figure S1.

**Figure 4:**
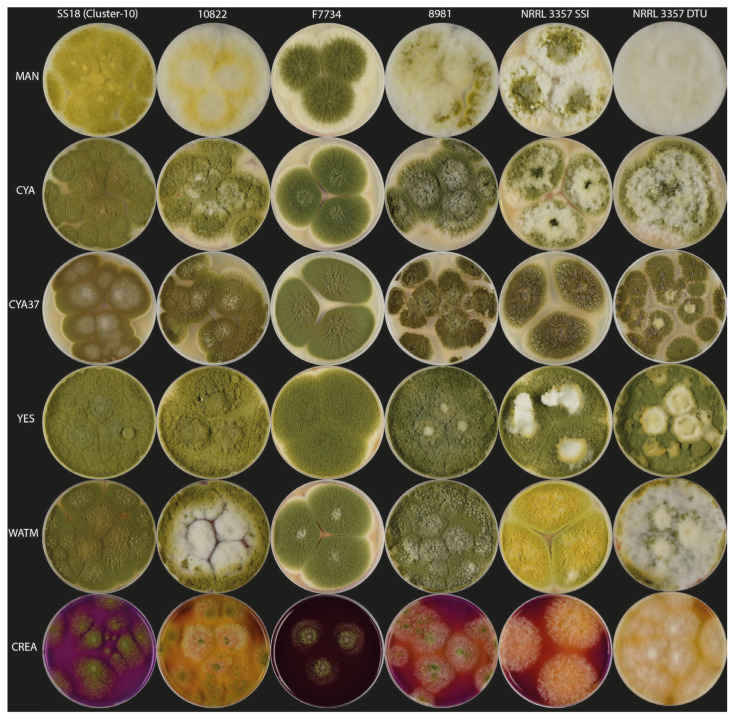
SS18 and main comparator morphology including two inter-laboratory NRRL 3357 isolates. The most prominent differences were observed on MAN, CYA, CYA37, YES, WATM, and CREA. For SS18 the lack of sclerotia on any media and lack of acid production on CREA was unique, whereas 10822 did not form sclerotia, but did produce acid while F7734 formed sclerotia (small glistening seeds) on CYA and WATM but had no acid production. 8981 stood out with its dark pigmentation, sulcate, suede-like appearance, especially on CYA37 and YES. No sclerotia were observed. NRRL 3357 isolates sourced from different laboratories showed many similarities on AFPA, CYA, CYA37, YES, MEAox, and SABO (here/Figure S1). Common for the two NRRL 3357 isolates (from the SSI and DTU collections, respectively) was fluffy white centres with pale-green conidiation in the margins and abundant sclerotia on CYA37 (brown/white dotted seeds). But more vegetative growth was observed on MAN, MEAox, and WATM for IBT 28520, and increased acid production on CREA.

The isolates showed substantial differences across the media, but most prominently on MAN, CYA, CYA37, YES, WATM, and CREA; see Figure 4.

Growth was comparable for SS18, 10822, and F7734 on DG18 media, but NRRL 3357 showed less growth and 8982 showed a vegetative cotton-like appearance.

Among isolates outside Cluster-10, eight formed sclerotia on WATM, seven on CYA, six on CYA37 and MEAox, and two on YES and OAT, see Figure 4 and Figure S1.

**Figure 5:**
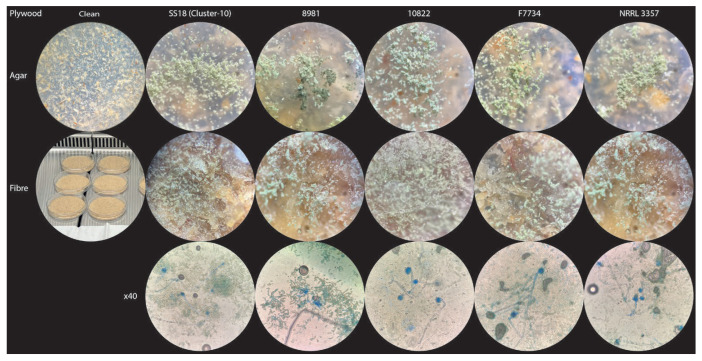
Growth of SS18 and main comparators on plywood-fibre agar and plywood-fibres directly. Despite small variations in colour patterns among strains, only 8981 appeared different with a dark green and patchy growth. Micromorphology: SS18: Conidiophore delicate, smooth, moderately thin walled, collapsed; vesicle clavate/globose; conidia globose, smooth, thick walled. 8981: Conidiophore smooth, moderately thick walled, distal branching; vesicles globose, small; conidia globose, rough with projections, moderately thick walled. 10822: Conidiophore smooth, moderately thick walled, collapsed; vesicles clavate/globose; conidia globose, smooth, thick walled. F7734: Conidiophore smooth, moderately thick walled, collapsed; vesicles clavate/globose; conidia globose, smooth, thick walled. NRRL 3357: Conidiophore smooth, moderately thick walled, collapsed; vesicles clavate/globose; conidia globose, smooth, moderately thick walled.

SS18 and the main comparators were further analysed for growth differences on plywood, which was a surface Cluster-10 isolates had previously shown active growth on inside the hospital [5]. All strains had comparable growth on plywood-fibre agar and on the plywood-fibres directly when wetted. Only slight colour variation and overall macro and micromorphology was observed among isolates except for 8981, which displayed darker, more patchy growth; see Figure 5.

### 3.3. Secondary Metabolite Profiling via HPLC-DAD

*A. flavus* is a rich producer of bioactive secondary metabolites, where some may play a role in virulence, adaptation with specific advantages, or as competitive traits for strain persistence. At DTU, HPLC-DAD is frequently used for routine analyses to examine metabolic responses of *A. flavus* in various conditions. The method can detect a number of bioactive molecules, and we hypothesised that the Cluster-10 isolates could display an altered profile given the specific monophyletic nature and unique morphotype. In this analysis, IDs of secondary metabolites were given via standardised retention indices from the internal DTU-library. UV-VIS chromatograms were compared to authentic standards and well-characterised chromatograms. Also, identified metabolites were compared to a previous study that characterised secondary metabolites from *A. flavus* (NRRL 3357) and *A. oryzae* (RIB40) via HPLC-DAD and LC-MS/MS [25]. Typical UV-VIS of the secondary metabolites presented here, retention indices distribution, retention times, and logA estimates are available in the Supplementary Files. An overview of metabolites detected in the isolates and their respective retention indices are provided in Table 3.

**Figure 6:**
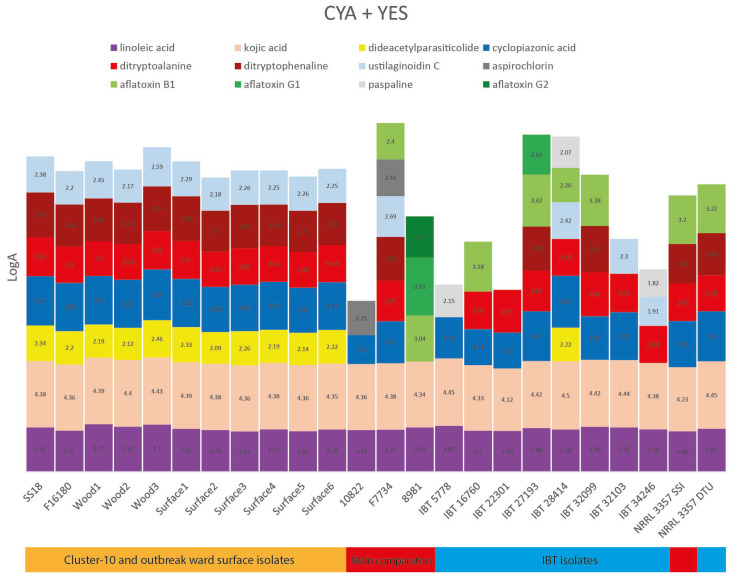
Secondary metabolite comparison from CYA + YES plugs combined for 24 *A. flavus* isolates. Detected secondary metabolites are displayed as log transformed (LogA) signal intensities expressed as area under the curve. Note that UV-absorption is metabolite specific, whereas intensity can only be compared across isolates for identical metabolites. The Cluster-10 isolates showed a distinct secondary metabolite profile with prominent signals for dideacetylparasiticolide, ustilaginoidin C, and absence of aflatoxin B_1_. Only one other (IBT 28414) produced dideacetylparasiticolide but aflatoxin B_1_ and paspaline was also detected in that isolate and the latter is found only in sclerotia which were prominent for IBT 28414 on the CYA medium (see Figure S1), but absent in Cluster-10 isolates. Notably, 8981 had intense signals for linoleic acid, kojic acid, aflatoxin B_1_, G_1_, and G_2_, but was lacking other secondary metabolites—a profile normally associated with *A. parasiticus*. The inter-laboratory NRRL 3357 controls had identical profiles. All isolates shared comparable signals for kojic acid and for the primary metabolism related fatty acid linoleic acid.

Secondary metabolite profiles were compared for combined plug-extracts from carbon rich CYA + YES media due to their promotion of a diverse secondary metabolism [28]. This showed a distinct chemical fingerprint for Cluster-10 isolates, different from the main comparators and the IBT isolates Figure 6.

**Figure 7:**
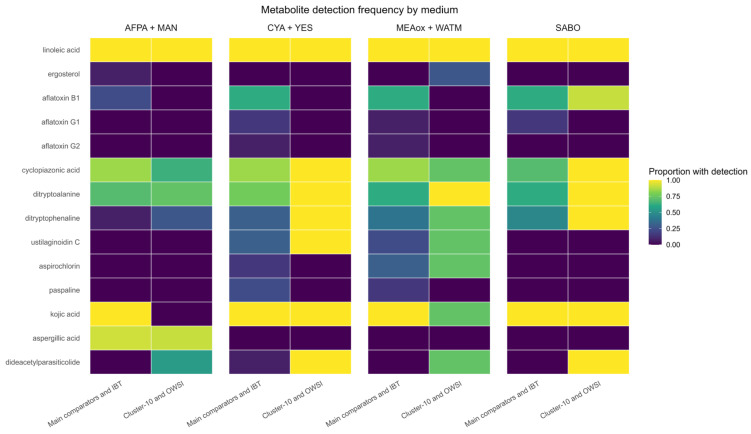
Secondary metabolite detection frequency across media for Cluster-10 and outbreak ward surface isolates (OWSI) in one group versus main comparators and IBT isolates (IBT) in the other. The secondary metabolite profile of Cluster-10 isolates and outbreak ward surface isolates were consistent on CYA + YES and MEAox + WATM comprising the combination of dideacetylparasiticolide, ustilaginoidin C, and absence of aflatoxin B_1_. However, no ustilaginoidin C was observed in AFPA + MAN, and aflatoxin B_1_ was present on SABO medium. Detection of aspergillic acid was observed only in AFPA + MAN for all isolates.

Although the Cluster-10 isolates did not produce aflatoxin B_1_ on the CYA + YES media, signals for aflatoxin B_1_ were detected on the SABO medium for most of them. Generally, the other aflatoxin producers showed enhanced aflatoxin signals on SABO. One additional compound was only detected in the AFPA + MAN plugs, aspergillic acid, and was produced by all isolates but one (Wood3). A heatmap displays metabolite profiles across different media; see Figure 7.

### 3.4. Genomic Basis of Secondary Metabolites

Representative BGCs from reference sequences available at NCBI for aflatoxin, cyclopiazonic acid, ditryptophenaline, and dideacetylparasiticolide were identified and compared for SS18 and the main comparators from whom short-read genomes were available from our previous study (ENA Acc. No. PRJEB105462) [5]. Prior to the analysis, sequences of ITS and *CaM* for 8981 were blasted to NCBI and both showed a 100% similarity to *A. parasiticus*. Consequently, this isolate was excluded from further gene cluster comparison, as it was probably a different species within *Aspergillus* section *Flavi*. Remaining isolates harboured BGCs with a high level of isogeneity to the BGCs of NRRL 3357 for aflatoxin, cyclopiazonic acid, and ditryptophenaline. The dideacetylparasiticolide gene cluster is defined in [27] and was examined due to this specific compound’s prominence in outbreak-related isolates. An intact BGC was found in SS18, whereas it was indeed partially absent in comparators, which did not produce the metabolite Table 4.

*A. flavus* strain E1404 [26] was included to verify the dideacetylparasiticolide clusters’ similarity to either E1404 or the query sequence from RIB40 (*A. oryzae*). Closest similarity was observed between SS18 and E1404 versus SS18 and RIB40, with 99.55% and 98.64% respectively.

## 4. Discussion

Intrigued by the notable dominance in the hospital environment and among patients in the outbreak ward, this study explored virulence in *Galleria mellonella*, morphological characteristics, secondary metabolite profiles and gene clusters for an *A. flavus* strain from a previously reported clonal outbreak [3,4,5]. No indication of increased virulence was observed among tested isolates, although increased mortality was indeed observed in larvae receiving a fungal strain versus pure PBS. Morphology on several culture agars had defined *A. flavus* features [29], but with a distinct and reproducible fingerprint morphotype for all isolates included in Cluster-10 (the monophyletic cluster with all outbreak isolates [5]). That was relative to genetically distinct isolates in the main comparators group and isolates from the IBT collection, which had not been sequenced. Similarly, the Cluster-10 isolates shared a uniform secondary metabolite pattern. The HPLC-DAD method yielded distinctive UV-VIS chromatograms that matched spectra and retention indices for LC-MS/MS characterised metabolites [25] identified here via authentic standards. For some metabolites, their detection was supported by the presence of BGCs. The Cluster-10 unique secondary metabolite profile comprised the combination of dideacetylparasiticolide [14,27] and ustilaginoidin C both produced by them all, and remarkably the lack of aflatoxin B_1_ production on all media except SABO. We speculate whether Cluster-10 dominance in the outbreak ward could be coupled to an overall unique phenotype defined for this monophyletic lineage.

The morphological development of *A. flavus* undergoes complex genetic regulation [18]. The overall macromorphology of Cluster-10 isolates was preserved across isolates. Prominent conidiation, lack of sclerotia, and low levels of acid production were some of their distinct features. The morphotype was reproducible, and that was also the case for the two inter-laboratory controls (NRRL 3357) on most media. Differences in NRRL 3357 morphology possibly reflected isolate fitness upon inoculation since their genotype and secondary metabolite profile were identical.

Conidia and sclerotia formation enable reproduction and perseverance in *A. flavus*. Common for both structures are their genetic regulation machinery [18,19]. Lack of sclerotial formation by Cluster-10 isolates could inversely reflect their ecological background indicating an evolutionary or regulatory trait that distances them from agricultural strains like NRRL 3357 that use sclerotia packed with aflatoxins to avoid predation by insects and preserve DNA during unfavourable environmental conditions [30]. Further investigation into regulatory genes could possibly determine which is more likely.

SS18 and the main comparators all grew on plywood substrates and had similar morphology except 8981 with patchy growth and notably dark coloration on several media. Of note, although 8981 was previously morphologically identified as *A. flavus* upon patient isolation, it showed ITS and *CaM* sequence homology with *A. parasiticus* here. This was supported by typical *A. parasiticus* morphology and a secondary metabolite profile with aflatoxin G_1_ and absence of cyclopiazonic acid [29]. Nonetheless, all STR loci in the STR*Afla* assay were previously amplified for isolate 8981 [3], implying homologous loci may be distributed across section *Flavi*. We previously verified STR*Afla* for reliable clonality detection with high discriminatory power and consistent identification of Cluster-10 isolates [5]. Thus, STR*Afla* can be used for clonality assessment in confirmed *A. flavus* outbreaks, provided species identification within section *Flavi* is independently verified by robust methods and not only determined by morphology. Interestingly, this *A. parasiticus* showed virulence comparable to both SS18 and the other main comparators in this study, although it seems rarely associated with human infection in the literature.

Secondary metabolite profiles of Cluster-10 isolates were mapped to authentic standards and showed reproducibility and narrow retention indices for prominent chromatograms of well-characterised *A. flavus* metabolites [25]. Similar reproducibility of secondary metabolite profile was observed for the two interlaboratory NRRL 3357 isolates compared to one another and to previous reports [25].

Contested microbiological environments like soil, fields, and human or animal tissues warrant antagonistic substances at disposal. Not surprisingly, some secondary metabolites have antimicrobial properties [31]. Application of non-aflatoxigenic *A. flavus* strains for crop biocontrol is a well-established method to curb contamination in agriculture [32]. Alshannaq et al. demonstrated that co-inoculation even at a 1:10 ratio effectively inhibits growth of aflatoxigenic *A. flavus* (NRRL 3357) strains [32]. Also, the filtrate alone from non-aflatoxigenic strains effectively inhibited growth of NRRL 3357 supporting a chemical aspect. However, these studies focused on aflatoxin mitigation via non-aflatoxigenic strains. In our study, aflatoxin B_1_ production by Cluster-10 isolates was only detected on Sabouraud medium, indicating a medium-dependent phenotype. Sabouraud medium is relatively simple, comprising a rich carbon source (glucose or dextrose) and a nitrogen source (peptone), while its pH is kept low at 5.6. The mechanism behind this Sabouraud-specificity for aflatoxin B_1_ in Cluster-10 isolates remains unknown, but other medium-dependent variations in aflatoxin biosynthesis have been described previously [33,34]. The nitrogen source, as an example, influences toxin production and gene-expression within the aflatoxin cluster in a strain-dependent manner [33]. Medium acidity is another modulator for some *A. flavus* strains [34]. 

Cluster-10 isolates dominated the affected ward [5], and hypothetically, a potential reason could include antagonism of other *A. flavus* strains. As an example, the chemical class of astellolides, that includes dideacetylparasiticolide, was stereotypic for Cluster-10 isolates, and other members of that class have previously shown potent antifungal effects with MICs reaching 1.56 mg/L for *Penicillium italicum* [14]. Hypothetically, the secondary metabolite profile of the Cluster-10 isolates could reflect adaptations to their combatant needs when they settle in specific environments. But that remains speculative. Future studies are needed for proper experimental testing of that hypothesis via MIC determination of potential antifungal compounds produced by Cluster-10 isolates and other inhibitory traits when co-cultured, or indeed by their filtrate directly added to colonies of other *A. flavus* strains [32].

A different aspect to mycotoxin presence in hospitals is the potential dangers of human exposure. Aflatoxins pose a well-known threat to humans when ingested, but there is limited evidence regarding other toxins like cyclopiazonic acid, which is known to be toxic to animals [10]. There is an ongoing debate as to whether mycotoxins contaminate indoor environments and whether that poses a realistic threat to human health. For mycotoxins to constitute a clinical challenge, patients must be exposed via ingestion or inhalation. However, Al Hallak et al. recently emphasised that the presence and aerosolization of mycotoxins are multifactorial. The level of fungal contamination on surfaces matters and varies greatly [13]. Studies show that a wide range of mycotoxins are indeed produced when moulds grow on interior building materials [35,36,37,38], and that surfaces can be contaminated with mycotoxins in moisture-damaged homes at quantities of 1.3 ppb in dust and 21–110 ppb on interior building materials [36]. Furthermore, Aleksic et al. demonstrated that the toxic aflatoxin-precursor, sterigmatocystin, is produced in quantities of 82.02–142.18 mg/m^2^ by *Aspergillus versicolor* when growing on wallpaper, but with aerosolisation of only 0.2% at an air velocity of 2 m/s [35]. Note that regular movement typically generates air velocities of approximately 0.3 m/s, whereas slamming doors can produce velocities of approximately 6 m/s [35]. Moularat et al. reported slightly higher sterigmatocystin aerosolisation of 1–4% depending on substrate (incl. wallpaper) [37]. Sterigmatocystin may not be a surrogate marker to infer aerosolisation of mycotoxins in general, though it supports that surface contamination and some aerosolisation of the mycotoxins reported here are possible. The outbreak ward surfaces initially contained a median of 800 (0–25,200) CFU/m^2^ (*A. flavus*) and 2400 (0–28,000) CFU/m^2^ (*Aspergillus* spp.) in total [5]. For context, NRRL 3357 can produce up to 200 ppb aflatoxin from 50 spores per mL cultured at 30 °C for 12 days in potato dextrose broth [32], whereas the WHO sets the limit for aflatoxins in food commodities at 0.5–15 ppb [39].

Examination of BGC similarities (length in base pairs and sequence similarities) supported the detected secondary metabolites in a manner of presence or absence and SS18 (Cluster-10) had intact aflatoxin, cyclopiazonic acid, ditryptophenaline, and dideacetylparasiticolide gene clusters. We previously speculated that the Cluster-10 isolates might be a subspecies different from other *A. flavus* [3], although subspecies and varieties are not accepted in *Aspergillus* taxonomic practice. That theory reflected the emphasis placed in literature on distinguishing *A. flavus* from *A. oryzae* [26,40,41], a domesticated, non-aflatoxigenic derivative of *A. flavus* used in the food industry. Specificity of current separation methods are debated [41]. Kim et al. proposed a phylogenetic approach combined with designation of functionally intact aflatoxin and cyclopiazonic acid gene clusters to determine *A. flavus* [26], whereas Han et al. were sceptical given the apparent non-systematic dissemination of accessory genes (secondary metabolite genes) among *A. flavus* and *A. oryzae* [41]. Cluster-10 isolates cannot decisively be designated as either at this point, but morphology and secondary metabolism point to *A. flavus* in this study, although the cluster may have emerged from a niche wild type background.

This study has several limitations. *Galleria mellonella* is a recognised model to study fungal virulence and the injection of approximately 3000 CFU per larva gave a sufficient response within a five-day scope in this study. However, only mortality was reported as standardisation of movement and melanisation assessment was too difficult [42] whereby potential valuable information was lost. Additionally, right-wing censoring was chosen due to cocoon formation by day six. Assay credibility could be verified by replications and additional approaches to virulence-testing could also be applied. Compound identification was based on comparison of retention indices and UV-VIS spectra with authentic standards of well-characterised *A. flavus* metabolites specified previously via LC-MS/MS by Rank et al., 2012 [25]. Minor deviations in retention index were within expected analytical variability. While this supports the proposed identifications, confirmation by an orthogonal technique such as LC-MS/MS was not performed in the present study and would provide additional structural specificity. BGC-comparisons were limited to sequence length and percentage identity relative to query sequences, however without assessment of gene integrity. Finally, none of the identified compounds were evaluated for bioactive properties, and any proposed ecological role in the persistence of the Cluster-10 isolates remains speculative.

In conclusion, this study did not identify a single factor explaining the prolonged dominance of the Cluster-10 isolates in the affected ward or its success in causing infection. Rather, two working hypotheses seem reasonable. (i) Introduction to the hospital via pre-contaminated wood material followed by reseeding of water damaged sites in the ward and prolonged dust-dwelling. Those scenarios were previously supported [5]. (ii) Furthermore, no evidence of increased virulence in *G. mellonella* was observed here, although that may not be reflective of human virulence. However, dominance in the outbreak ward may not be virulence driven. The Cluster-10 isolates exhibited a distinct and reproducible phenotype. This included production of metabolites that could be further studied to determine whether the phenotype of Cluster-10 isolates confers fitness advantages over competing *A. flavus* strains. In addition, Cluster-10 isolates produced several poisonous mycotoxins with potential implications for human health, which also calls for further investigation in future studies.

Determinants of ward-dominance, which likely explain the *A. flavus* outbreak among patients, remain unclear, but this study highlighted potential features to study the success of Cluster-10 isolates that could prove useful in future *A. flavus* hospital outbreaks.

## Figures and Tables

**Table 1:** Overview of comparator isolates and available metadata.

Isolates (Local Name and Info.)	Origin (Original Strain Number)	Sourced from	Material	Year	SNPs from SS18
**Previously Sequenced [5]**					
**Cluster-10 isolates**					
SS18	DK	Outbreak ward surface	Dust (Vacuum cleaner bag)	2024	(Ref.)
F16180	DK	DCM patient	Sputum	2023	76
Wood1	Unknown	Danish retailer	Oriented strand board (OSB)	2020	82
Wood2	Unknown	Danish retailer	Medium-density fibreboard (MDF)	2020	86
Wood3	Unknown	Danish retailer	Plywood	2020	82
**Main comparators**					
10822	DK	Non-outbreak patient	BAL	2019	16,825
F7734	DK	Non-outbreak patient	BAL	2023	47,448
8981	DK	Non-outbreak patient	BAL	2018	Not in tree ^b^
NRRL 3357 SSI ^a^	USA (CBS 128202)	Statens Serum Institut (DK)	Peanut	1968	50,462
**Non-sequenced**					
**Outbreak ward surface isolates**					
Surface 1 to 6	DK	Outbreak ward surfaces	Surface swabs	2025	
**IBT isolates**					
IBT 5778	USA (NRRL 1957)	Danish Technical University	Cellophane	1944	
IBT 16760	Ecuador	Danish Technical University	Soil	1965	
IBT 22301	Finland	Danish Technical University	Building material	Unknown	
IBT 27193	The Netherlands	Danish Technical University	Unknown	Unknown	
IBT 28414	DK	Danish Technical University	Lake water	2006	
IBT 32099	USA—Miami (SRRC 2653)	Danish Technical University	Corneal ulcer	Unknown	
IBT 32103	USA—Chicago (SRRC 2632)	Danish Technical University	Clinical (unknown site)	Unknown	
IBT 34246	DK	Danish Technical University	Wheat	Unknown	
NRRL 3357 DTU ^a^	USA (NRRL 3357)	Danish Technical University	Peanut	1968	

^a^ NRRL 3357 SSI and NRRL 3357 DTU was the same strain, but sourced from SSI and DTU respectively. Both had identical genotypes in all tested short tandem repeat genotype markers (6/9). STR*Afla*-markers 2A-3C: 15;16;20;19;16;14. ^b^ 8981 was previously sequenced but excluded from phylogeny due to few callable positions [5]. SNPs: Single Nucleotide Polymorphisms, BAL: Bronchoalveolar lavage, DK: Denmark, USA: United States of America.

**Table 2:** Survival time in days for all larval groups infected by SS18 or the main comparators.

Strain	RMST (Days)	Difference (Days)	CI	*p* ^a^
SS18 (Cluster-10)	2.50	(Ref.)	-	-
NRRL 3357	2.30	−0.20	[−1.00; 0.55]	0.68
10822	2.35	−0.15	[−1.05; 0.75]	0.78
8981	2.85	0.35	[−0.65; 1.35]	0.50
F7734	3.60	1.10	[0.10; 2.05]	0.03

^a^ After BH-adjustment all *p*-values were >0.05.

**Table 3:** Detection of secondary metabolites in 24 isolates, including associated retention index standards and ranges.

Metabolite IDs	No. Isolates with Detection	Retention Index (RI)	RI Range
Kojic acid *	24	558	556–560
Ustilaginoidin C **	17	849	847–849
Ditryptoalanine ***	19	952	942–959
Ditryptophenaline ***	23	967	958–969
Aflatoxin G_1_ *	2	815	815–820
Aflatoxin G_2_ *	1	712	
Aflatoxin B_1_ *	18	840	839–845
Aspirochlorin ***	13	857	850–860
Dideacetylparasiticolide ***	13	1065	1065, 1066
Cyclopiazonic acid ***	22	1099	1097–1103
Paspaline ***	4	1486	1485–1486
Linoleic acid * (in all fungi)	24	1615	1614–1619
Aspergillic acid *	22	1986	1984–1988
Ergosterol * (in all fungi)	4	2135	2126–2138

* Compared with an in-house authentic standard. ** Based on a very unique UV spectrum. *** Compared with an authentic standard and previously characterised in NRRL 3357 or RIB40 via LC-MS/MS (Rank et al. 2012) [25].

**Table 4:** Aflatoxin, cyclopiazonic acid, ditryptophenaline, and dideacetylparasiticolide BGC sizes and identity among SS18 and main comparator isolates compared to query sequences from NRRL 3357, RIB40, or E1404.

Toxin/Metabolite	Strain	Seq. Size (bp)	Identity (%)
Aflatoxin	NRRL 3357 (NCBI ref. sequence)	70,001	(Ref)
	SS18 (Cluster-10)	69,706	97.69
	10822	69,732	97.63
	F7734	69,763	98.84
Cyclopiazonic acid	NRRL 3357	24,262	(Ref)
	SS18	20,029	78.67
	10822	21,344	83.44
	F7734	24,269	99.02
Ditryptophenaline	NRRL 3357	14,001	(Ref)
	SS18	13,982	99.59
	10822	13,975	99.71
	F7734	13,992	99.93
Dideacetylparasiticolide	RIB40 (*A. oryzae* NCBI ref. sequence)	16,857	(Ref)
	SS18	16,872	98.64
	10822	1725	9.38
	F7734	1725	9.40
	NRRL 3357	1725	9.40
	E1404 (*A. flavus* NCBI ref. sequence)	16,872	98.99
	E1404 vs. SS18	-	99.55

## Data Availability

Genomic data used are publicly available from the following ENA genomic sequence accession number: PRJEB105462.

## Supplementary Materials

The following supporting information can be downloaded at: https://www.mdpi.com/article/10.3390/jof12060454/s1, Figure S1: Morphology of all strains; Figure S2: UV-VIS chromatograms; Table S1: HPLC-DAD data.

## References

[1] Hedayati M.T., Pasqualotto A.C., Warn P.A., Bowyer P., Denning D.W. (2007). *Aspergillus flavus*: Human pathogen, allergen and mycotoxin producer. Microbiology.

[2] Vonberg R.-P., Gastmeier P. (2006). Nosocomial aspergillosis in outbreak settings. J. Hosp. Infect..

[3] Gewecke A., Hare R.K., Salgård C., Kyndi L., Høg M., Petersen G., Nahimana D., Abou-Chakra N., Knudsen J.D., Rosendahl S. (2024). A single-source nosocomial outbreak of *Aspergillus flavus* uncovered by genotyping. Microbiol. Spectr..

[4] Vissing N.H., Lausen B., Hutchings Hoffmann M., Als-Nielsen B., Schmiegelow K., Helweg-Larsen J., Arendrup M.C., Nygaard U. (2021). *Aspergillus flavus* Infections in Children with Leukemia despite Liposomal Amphotericin-B Prophylaxis. Pediatr. Infect. Dis. J..

[5] Gewecke A., Sieber R.N., Hallstrøm S., Stegger M., Kolarik B., Knudsen J.D., Andersen B., Hall A., Vissing N.H., Arendrup M.C. (2026). Environmental and Phylogenetic Investigations of *Aspergillus flavus* Outbreak Linked to Contaminated Building Materials, Denmark, 2025. Emerg. Infect. Dis..

[6] Loukou E., Jensen N.F., Rohde L., Andersen B. (2026). Pre-contamination of new, wood-based building materials: Fungal diversity and susceptibility under different moisture scenarios. Build. Environ..

[7] Yohannis E., Urugo M.M., Teka T.A., Getachew P., Tola Y.B., Forsido S.F., Kebede Y.S., Teferra T.F. (2025). Aflatoxin Contamination in Agri-Food Systems: A Comprehensive Review of Toxicity, Food Security, Economic Impacts, and Sustainable Mitigation Across the Value Chain. Food Sci. Nutr..

[8] Pickova D., Ostry V., Toman J., Malir F. (2021). Aflatoxins: History, Significant Milestones, Recent Data on Their Toxicity and Ways to Mitigation. Toxins.

[9] Goessens T., Tesfamariam K., Njobeh P.B., Matumba L., Jali-Meleke N., Gong Y.Y., Herceg Z., Ezekiel C.N., De Saeger S., Lachat C. (2025). Incidence and mortality of acute aflatoxicosis: A systematic review. Environ. Int..

[10] Ostry V., Toman J., Grosse Y., Malir F. (2018). Cyclopiazonic acid: 50th anniversary of its discovery. World Mycotoxin J..

[11] Valdes J.J., Cameron J.E., Cole R.J. (1985). Aflatrem: A tremorgenic mycotoxin with acute neurotoxic effects. Environ. Health Perspect..

[12] Feng M.L., Li Z.H., Shi B.B. (2025). Progress on 3-Nitropropionic Acid Derivatives. Biomolecules.

[13] Al Hallak M., Verdier T., Bertron A., Roques C., Bailly J.D. (2023). Fungal Contamination of Building Materials and the Aerosolization of Particles and Toxins in Indoor Air and Their Associated Risks to Health: A Review. Toxins.

[14] Chen T., Yang W., Li T., Yin Y., Liu Y., Wang B., She Z. (2022). Hemiacetalmeroterpenoids A-C and Astellolide Q with Antimicrobial Activity from the Marine-Derived Fungus *Penicillium* sp. N-5. Mar. Drugs.

[15] Dutcher J.D. (1947). Aspergillic Acid: An Antibiotic Substance Produced by *Aspergillus flavus*: I. General Properties; Formation Of Desoxyaspergillic Acid; Structural Conclusions. J. Biol. Chem..

[16] Zhang Q., Jia M., Li H., Shi T., Xu Y., Zhao T., Zhang L., Zhao P., Xia X. (2025). Epipolythiodioxopiperazines: From Chemical Architectures to Biological Activities and Ecological Significance—A Comprehensive Review. Fermentation.

[17] Balbinot R.B., de Oliveira J.A.M., Bernardi D.I., Polli A.D., Polonio J.C., Cabral M.R.P., Zanqueta É.B., Endo E.H., Meneguello J.E., Cardoso R.F. (2021). *Chromolaena laevigata* (Asteraceae) as a source of endophytic non-aflatoxigenic *Aspergillus flavus*: Chemical profile in different culture conditions and biological applications. Braz. J. Microbiol..

[18] Calvo A.M., Cary J.W. (2015). Association of fungal secondary metabolism and sclerotial biology. Front. Microbiol..

[19] Cho H.J., Son S.H., Chen W., Son Y.E., Lee I., Yu J.H., Park H.S. (2022). Regulation of Conidiogenesis in *Aspergillus flavus*. Cells.

[20] Firacative C., Khan A., Duan S., Ferreira-Paim K., Leemon D., Meyer W. (2020). Rearing and Maintenance of *Galleria mellonella* and Its Application to Study Fungal Virulence. J. Fungi.

[21] Han K., Jung I. (2022). Restricted Mean Survival Time for Survival Analysis: A Quick Guide for Clinical Researchers. Korean J. Radiol..

[22] Iwasaki T., Kosikowski F.V. (1973). PRODUCTION OF β-NITROPROPIONIC ACID IN FOODS. J. Food Sci..

[23] Smedsgaard J. (1997). Micro-scale extraction procedure for standardization screening of fungal metabolite production in cultures. J. Chromatogr. A.

[24] Frisvad J.C., Thrane U. (1987). Standardized high-performance liquid chromatography of 182 mycotoxins and other fungal metabolites based on alkylphenone retention indices and UV-VIS spectra (diodearray detection). J. Chromatogr. A.

[25] Rank C., Klejnstrup M.L., Petersen L.M., Kildgaard S., Frisvad J.C., Held Gotfredsen C., Ostenfeld Larsen T. (2012). Comparative Chemistry of *Aspergillus oryzae* (RIB40) and A. flavus (NRRL 3357). Metabolites.

[26] Kim D.H., Kim D.C., Seo D., Kim K.T., Lee S.H., Hong S.B. (2025). Genome diversity, population structure and MALDI-TOF MS profiling of *Aspergillus oryzae*/flavus strains from fermentation and wild environments. BMC Genom..

[27] Shinohara Y., Takahashi S., Osada H., Koyama Y. (2016). Identification of a novel sesquiterpene biosynthetic machinery involved in astellolide biosynthesis. Sci. Rep..

[28] Samson R.A., Visagie C.M., Houbraken J., Hong S.B., Hubka V., Klaassen C.H.W., Perrone G., Seifert K.A., Susca A., Tanney J.B. (2014). Phylogeny, identification and nomenclature of the genus Aspergillus. Stud. Mycol..

[29] Frisvad J.C., Hubka V., Ezekiel C.N., Hong S.B., Nováková A., Chen A.J., Arzanlou M., Larsen T.O., Sklenář F., Mahakarnchanakul W. (2019). Taxonomy of Aspergillus section Flavi and their production of aflatoxins, ochratoxins and other mycotoxins. Stud. Mycol..

[30] Rohlfs M., Churchill A.C.L. (2011). Fungal secondary metabolites as modulators of interactions with insects and other arthropods. Fungal Genet. Biol..

[31] Wang S., Wang Y., Shi X., Herrera-Balandrano D.D., Chen X., Liu F., Laborda P. (2024). Application and antagonistic mechanisms of atoxigenic Aspergillus strains for the management of fungal plant diseases. Appl. Environ. Microbiol..

[32] Alshannaq A.F., Gibbons J.G., Lee M.K., Han K.H., Hong S.B., Yu J.H. (2018). Controlling aflatoxin contamination and propagation of *Aspergillus flavus* by a soy-fermenting *Aspergillus oryzae* strain. Sci. Rep..

[33] Ehrlich K.C., Cotty P.J. (2002). Variability in nitrogen regulation of aflatoxin production by *Aspergillus flavus* strains. Appl. Microbiol. Biotechnol..

[34] Ehrlich K.C., Montalbano B.G., Cotty P.J. (2005). Divergent regulation of aflatoxin production at acidic pH by two Aspergillus strains. Mycopathologia.

[35] Aleksic B., Draghi M., Ritoux S., Bailly S., Lacroix M., Oswald I.P., Bailly J.D., Robine E. (2017). Aerosolization of Mycotoxins after Growth of Toxinogenic Fungi on Wallpaper. Appl. Environ. Microbiol..

[36] Täubel M., Sulyok M., Vishwanath V., Bloom E., Turunen M., Järvi K., Kauhanen E., Krska R., Hyvärinen A., Larsson L. (2011). Co-occurrence of toxic bacterial and fungal secondary metabolites in moisture-damaged indoor environments. Indoor Air.

[37] Moularat S., Robine E. (2008). A Method to Determine the Transfer of Mycotoxins from Materials to Air. CLEAN—Soil Air Water.

[38] Gutarowska B., Sulyok M., Krska R. (2010). A study of the toxicity of moulds isolated from dwellings. Indoor Built Environ..

[39] Mycotoxins. https://www.who.int/news-room/fact-sheets/detail/mycotoxins.

[40] Chang P.K. (2019). Genome-wide nucleotide variation distinguishes *Aspergillus flavus* from *Aspergillus oryzae* and helps to reveal origins of atoxigenic A. flavus biocontrol strains. J. Appl. Microbiol..

[41] Han D.M., Baek J.H., Choi D.G., Jeon M.-S., Eyun S.-I., Jeon C.O. (2024). Comparative pangenome analysis of *Aspergillus flavus* and *Aspergillus oryzae* reveals their phylogenetic, genomic, and metabolic homogeneity. Food Microbiol..

[42] Champion O.L., Titball R.W., Bates S. (2018). Standardization of G. mellonella Larvae to Provide Reliable and Reproducible Results in the Study of Fungal Pathogens. J. Fungi.

