# Supplementary material for: Distinct Phenotype and Secondary Metabolite Profile Mark a Dominant Aspergillus flavus Outbreak Strain
Source: J Fungi (Basel). 2026 Jun 22;12(6):454. doi: 10.3390/jof12060454 (PMC13301369; doi:10.3390/jof12060454)
Supplement: Supplementary file 1 [file jof-12-00454-s001.zip › Figure S2 UV VIS chromatograms.pdf]

UV-VIS of tentative secondary metabolites (including linoleic acid and ergosterol)

Approximate alkylphenone standard retention times (minutes): 7.12, 9.67, 11.57, 13.20, 14.67, 17.25, 19.42

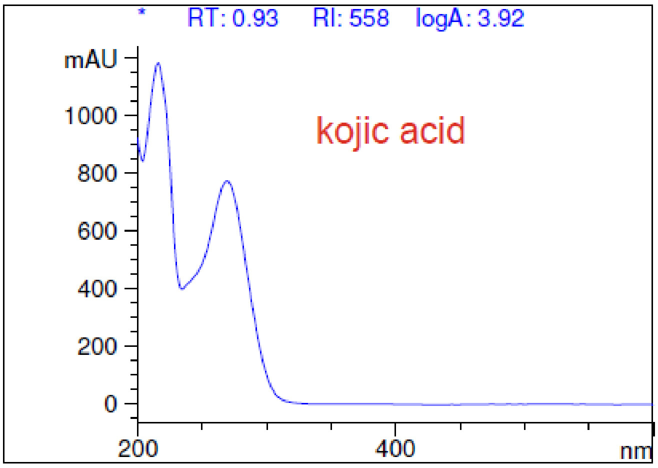

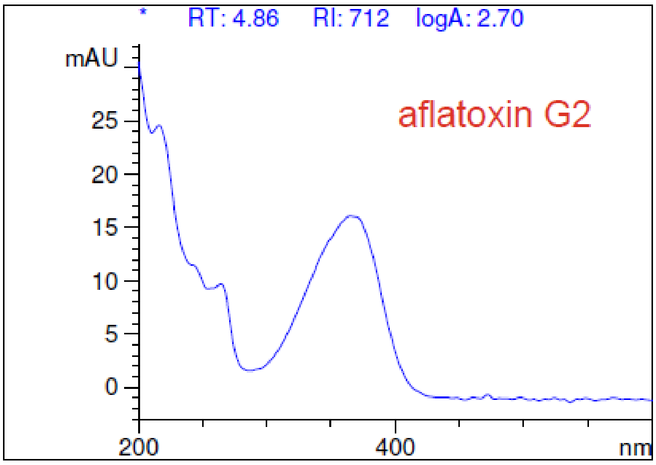

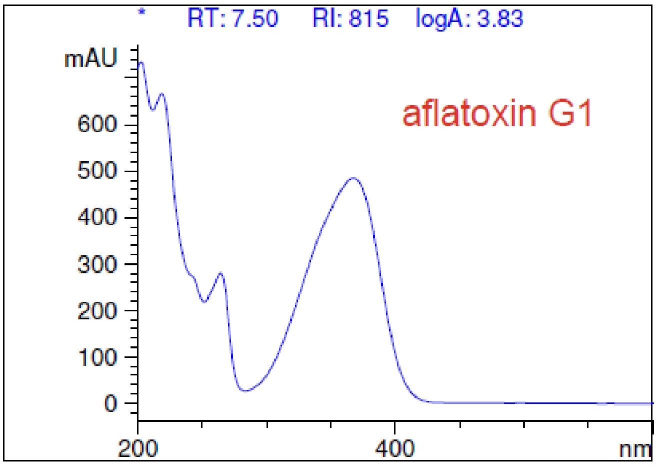

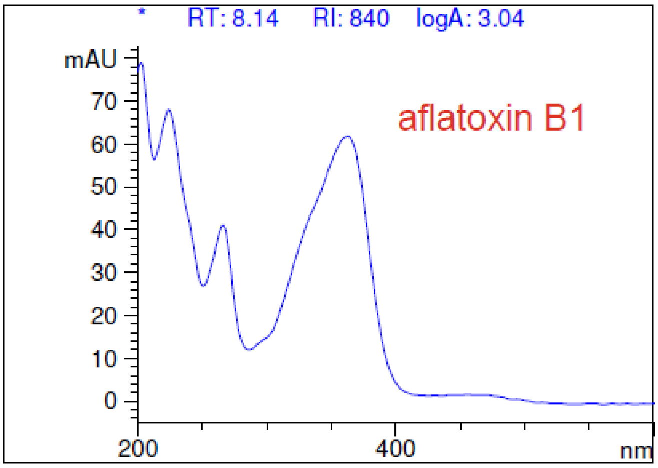
* RT: 8.14 RI: 840 logA: 3.04
mAU
70
60
50
40
30
20
10
0
200
400
nm
aflatoxin B1

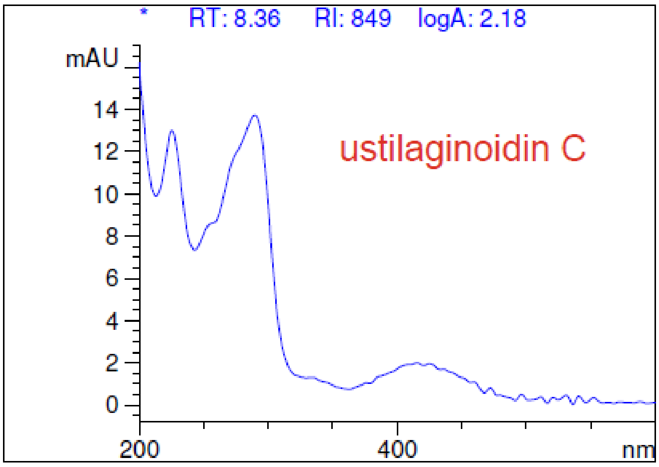
* RT: 8.36 RI: 849 logA: 2.18
mAU
14
12
10
8
6
4
2
0
200
400
nm
ustilaginoidin C

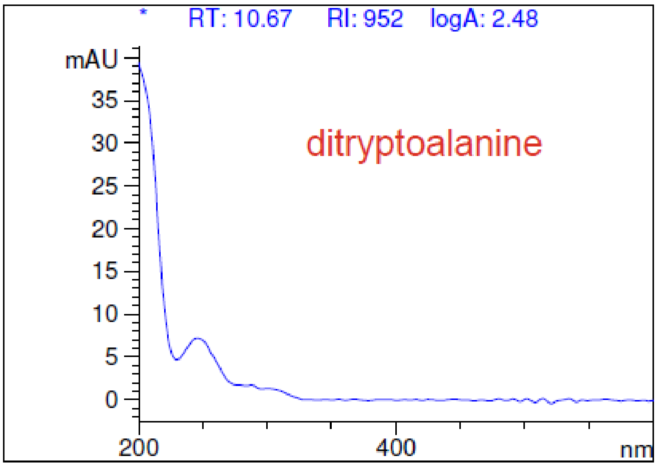
* RT: 10.67 RI: 952 logA: 2.48
mAU
35
30
25
20
15
10
5
0
200
400
nm
ditryptoalanine

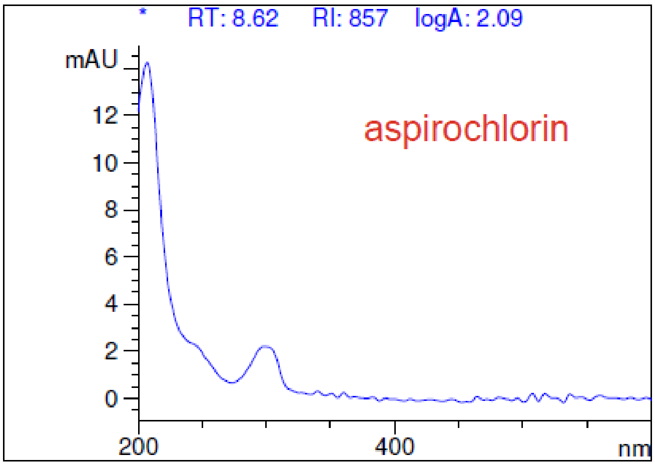

*
RT: 8.62
RI: 857
logA: 2.09
mAU
12
10
8
6
4
2
0
aspirochlorin
200
400
nm

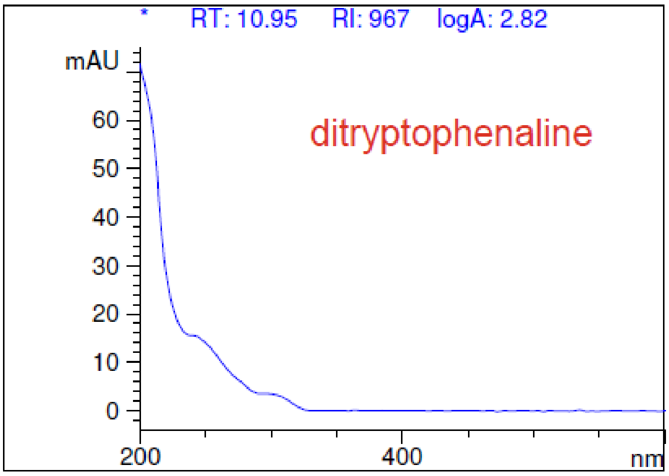

*
RT: 10.95
RI: 967
logA: 2.82
mAU
60
50
40
30
20
10
0
ditryptophenaline
200
400
nm

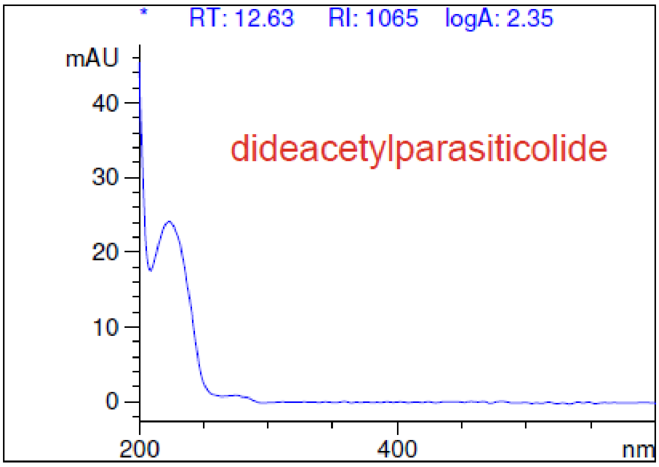

*
RT: 12.63
RI: 1065
logA: 2.35
mAU
40
30
20
10
0
dideacetylparasiticolide
200
400
nm

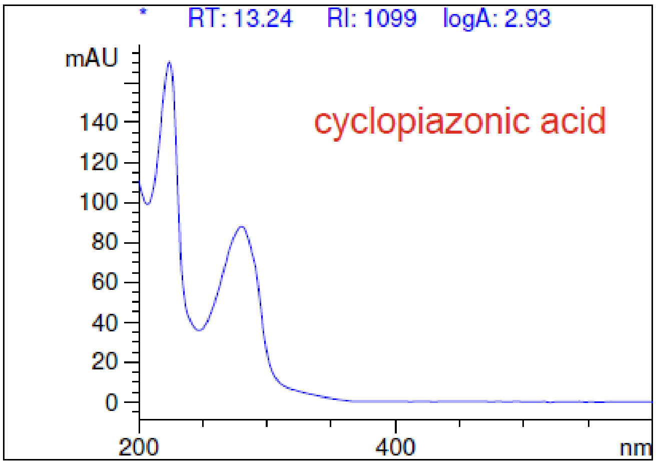
* RT: 13.24 RI: 1099 logA: 2.93
mAU
140
120
100
80
60
40
20
0
200
400
nm
cyclopiazonic acid

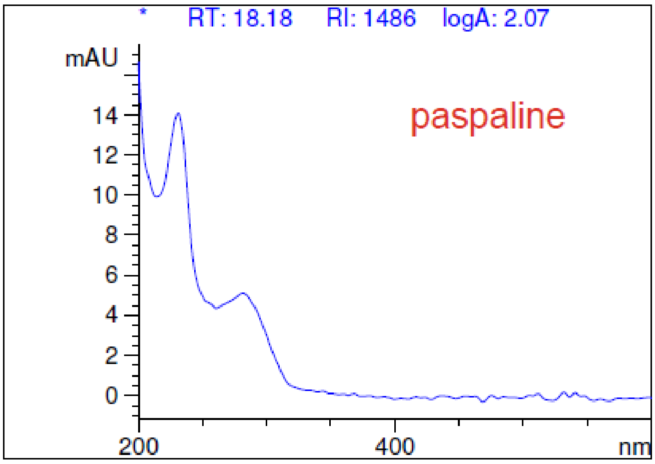
* RT: 18.18 RI: 1486 logA: 2.07
mAU
14
12
10
8
6
4
2
0
200
400
nm
paspaline

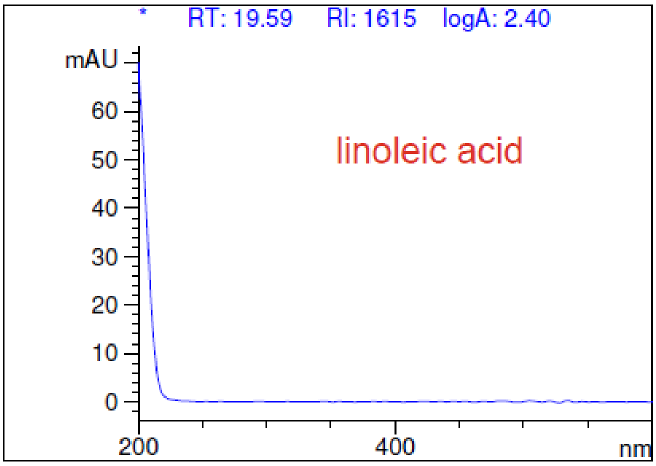
* RT: 19.59 RI: 1615 logA: 2.40
mAU
60
50
40
30
20
10
0
200
400
nm
linoleic acid

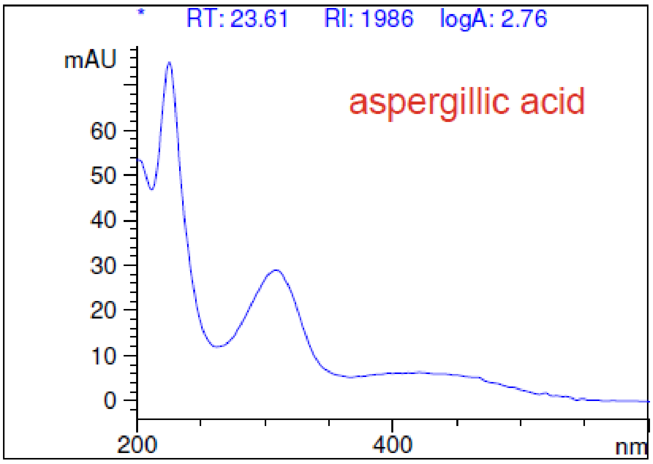
* RT: 23.61 RI: 1986 logA: 2.76
mAU
60
50
40
30
20
10
0
200
400
nm
aspergillic acid

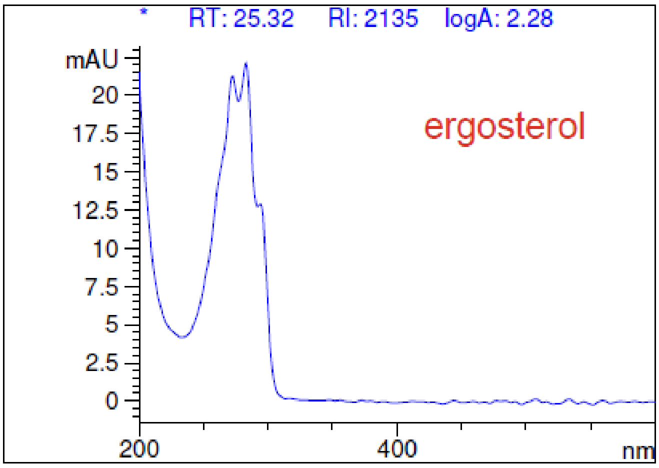
* RT: 25.32 RI: 2135 logA: 2.28
mAU
20
17.5
15
12.5
10
7.5
5
2.5
0
200
400
nm
ergosterol
