# Supplementary figures and images for: Distinct Phenotype and Secondary Metabolite Profile Mark a Dominant Aspergillus flavus Outbreak Strain
Source: J Fungi (Basel). 2026 Jun 22;12(6):454. doi: 10.3390/jof12060454 (PMC13301369; doi:10.3390/jof12060454)

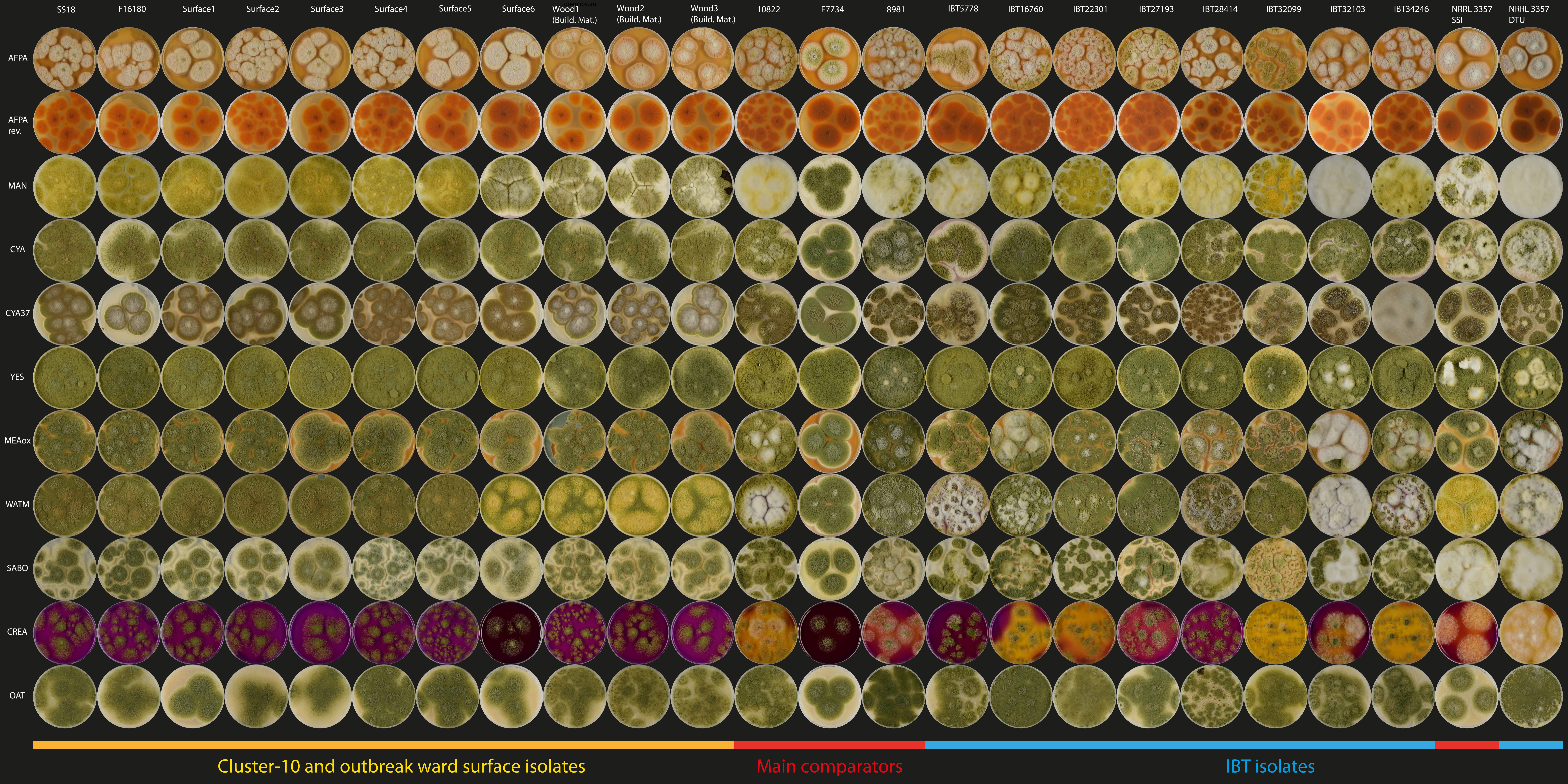

Supplement: Supplementary file 1 [file jof-12-00454-s001.zip › Figure S1 Morphology of all strains.pdf]
